# Lung Field Segmentation in Chest X-ray Images Using Superpixel Resizing and Encoder–Decoder Segmentation Networks

**DOI:** 10.3390/bioengineering9080351

**Published:** 2022-07-29

**Authors:** Chien-Cheng Lee, Edmund Cheung So, Lamin Saidy, Min-Ju Wang

**Affiliations:** 1 Department of Electrical Engineering, Yuan Ze University, Taoyuan 320, Taiwan; lsaidy9@gmail.com; 2 Department of Anesthesia, An Nan Hospital, China Medical University, Tainan 709, Taiwan; edmundsotw@gmail.com; 3 Department of Radiology, An Nan Hospital, China Medical University, Tainan 709, Taiwan; t70213@mail.tmanh.org.tw

**Keywords:** lung segmentation, encoder–decoder network, superpixels, downsampling interpolation, upsampling interpolation

## Abstract

Lung segmentation of chest X-ray (CXR) images is a fundamental step in many diagnostic applications. Most lung field segmentation methods reduce the image size to speed up the subsequent processing time. Then, the low-resolution result is upsampled to the original high-resolution image. Nevertheless, the image boundaries become blurred after the downsampling and upsampling steps. It is necessary to alleviate blurred boundaries during downsampling and upsampling. In this paper, we incorporate the lung field segmentation with the superpixel resizing framework to achieve the goal. The superpixel resizing framework upsamples the segmentation results based on the superpixel boundary information obtained from the downsampling process. Using this method, not only can the computation time of high-resolution medical image segmentation be reduced, but also the quality of the segmentation results can be preserved. We evaluate the proposed method on JSRT, LIDC-IDRI, and ANH datasets. The experimental results show that the proposed superpixel resizing framework outperforms other traditional image resizing methods. Furthermore, combining the segmentation network and the superpixel resizing framework, the proposed method achieves better results with an average time score of 4.6 s on CPU and 0.02 s on GPU.

## 1. Introduction

Chest X-ray (CXR) is the most common imaging technique widely used for lung diagnosis and treatment, especially for COVID-19. Lung segmentation of CXR images is a fundamental step in many diagnostic applications involving the detection, recognition, and analysis of anatomical structures in computer-aided diagnosis systems. However, manual identification of lung fields is time-consuming and error prone. Thus, accurate automatic segmentation of lung fields has received attention from researchers as an essential preprocessing step in automatically analyzing chest radiographs.

CXR images have higher resolutions. For example, the resolution of the public Japanese Society of Radiological Technology (JSRT) dataset [1] is 2048 × 2048, which is widely used to evaluate the performance of CXR lung segmentation methods. Therefore, most CXR lung field segmentation studies downsample the image size to 128 × 128 or 256 × 256 through linear interpolation to reduce the computation time [2,3,4], especially for deep learning-based methods. However, during downsampling, the graylevel information of several pixels in the high-resolution images is merged to form the graylevel information of a pixel in the downsampled low-resolution images. Thus, the boundary information loss of pixels is unavoidable and causes boundary blurs or missing of the low-resolution images. On the other hand, most methods of lung segmentation usually rely on large gray value contrasts between lung fields and surrounding tissues. As a result, the quality of the segmentation for the image with blurred boundaries will degrade.

Additionally, high-resolution images are necessary for practical medical applications. Thus, the segmentation methods based on downsampling preprocessing need to upsample the segmented results to the original high-resolution images. However, the upsampled results obtained from pixels of the low-resolution images will contain artifacts. Without sufficient information, the boundaries of segmented tissues are hard to correctly recover during upsampling. As a result, the quality of the upsampled segmentation results is worse than that of the results processed from the original high-resolution images.

Solving the downsampling blurred boundary problem and the upsampling artifact problem is necessary for deep learning-based segmentation methods for high-resolution medical images. Most existing stand-alone downsampling and upsampling methods, such as bilinear, bicubic, and nearest neighbor interpolation algorithms, focus on image downsampling and upsampling independent entities, rather than coupling steps simultaneously. Thus, the existing methods cannot solve the problems mentioned above. To alleviate these problems, this study employs a superpixel resizing framework to reduce information loss during downsampling and reconstruct the boundaries of foreground segmentation results during upsampling.

This paper proposes a lung field segmentation combining the ultrafast superpixel extraction via quantization (USEQ) [5] superpixel resizing framework. Using this method can reduce the computational time of high-resolution medical image segmentation and preserve the quality of the segmentation results. In the experimental results, three datasets are used to demonstrate the segmentation performance of the proposed method. Furthermore, we evaluate the performance of USEQ resizing and the bicubic interpolation resizing algorithms in downsampling and upsampling steps. Lung field segmentation results using the USEQ superpixel resizing framework significantly outperform other stand-alone resizing methods.

The remainder of this paper is organized as follows: Section 2 presents related work. Section 3 describes the datasets and critical components of our proposed method. We present our experimental results as well as an analysis in Section 4. Finally, Section 5 concludes this paper.

## 2. Related Work

In this section, we briefly revisit recent works on lung field segmentation and superpixel algorithms.

### 2.1. Lung Field Segmentation

Over the past decades, several lung field segmentation methods have been proposed. Hu et al. [6] proposed a three-step process of identifying the lungs in three-dimensional pulmonary X-ray CT (computed tomography) images. Additionally, Wang et al. [7] also used a three-step approach to segmenting lungs with severe interstitial lung disease (ILD) in thoracic CT. Alternatively, a fuzzy-based automatic lung segmentation technique from CT images was proposed [8]. This system needs no prior assumption of images. Sluimer et al. [9] proposed a refined segmentation-by-registration scheme based on an atlas to segment the pathological lungs in CT. Chama et al. [10] introduced an improved lung field segmentation in CT using mean shift clustering. Ibragimov et al. [11] used a supervised landmark-based segmentation in CXR lung field segmentation. Similarly, Yang et al. [4] proposed a computationally efficient method of lung field segmentation using structured random forests to detect lung boundaries from CXR images. Their approach is highly computationally efficient; it promotes a fast and practical procedure of lung field segmentation.

Deformable model-based methods adopt the internal force from object shape and the external force from image appearance to guide the lung segmentation. Back in 2006, Van Ginneken et al. [12] compared three methods for segmenting the lung fields in CXRs, including active shape model (ASM), active appearance model (AAM), and pixel classification. A hierarchical deformable approach based on shape and appearance models originated from the work conducted by Shao et al. [13]. Similarly, learnable MGRF (Markov–Gibbs random field) was introduced by Soliman et al. to accurately segment pathological and healthy lungs for reliable computer-aided disease diagnostics [14]. Their module integrates two visual appearance sub-models with an adaptive lung shape sub-model. A fully automated approach to segmenting lungs with high-density pathologies has been introduced [15]. They utilized a novel robust active shape model matching method to roughly segment the lungs’ outline. Hu and Li [16] proposed an automatic segmentation method of lung field in CXRs based on the improved Snake model. Bosdelekidis and Ioakeimidis [17] introduced a deformation-tolerant procedure based on approximating rib cage seed points for lung field segmentation.

Deep learning is state-of-the-art in semantic image segmentation [18,19,20,21,22]. Novikov et al. [3] proposed convolutional neural network (CNN) architectures for automated multi-class segmentation of lungs, clavicles, and heart on a dataset. Most deep learning segmentation algorithms adapt an encoder–decoder architecture, e.g., U-net and Seg-Net [23,24]. U-Net is an encoder–decoder network model that has served as the baseline architecture for most CXR segmentation models. Many studies have tried to modify the U-Net structure. For example, Wang [2] used a U-Net to segment multiple anatomical structures in CXRs. Arora et al. [25] proposed a modified UNet++ framework, and Yahyatabar et al. [26] offered a Dense-Unet inspired by DenseNet and U-Net for the segmentation of lungs. Moreover, Wang et al. [27] proposed a cascaded learning framework for the automated detection of pneumoconiosis, including a machine learning-based pixel classifier for lung field segmentation, and Cycle-Consistent Adversarial Networks (CycleGAN0) for generating large lung field images for training, and a CNN-based image classier.

### 2.2. Superpixels

A superpixel is a group of perceptually similar pixels. Superpixels represent image regions and adhere to intensity edges for segmentation purposes. There are three main desirable properties for superpixel extraction algorithms [28]: (1) superpixels should accurately adhere to image boundaries and should consist of perceptually similar pixels; (2) superpixels should be computationally efficient as they are used in preprocessing and postprocessing steps; (3) superpixels should improve speed and segmentation quality.

Superpixel algorithms are divided into two categories: graph-based and gradient-ascent-based methods. Graph-based methods treat each pixel as a node in the graph and the edge weights between two nodes are proportional to the similarity between neighboring pixels. The superpixels are generated by minimizing a cost function defined over the graph [29,30,31,32]. Gradient-ascent-based methods [28,33,34,35,36] start from a rough initial clustering of pixels and apply gradient-ascent methods. It takes steps proportional to the positive of the gradient and approaches a local maximum of that function. Then, it iteratively refines the clusters until some convergence criterion is met to form superpixels.

Compared to the superpixel mentioned earlier, the USEQ algorithm achieves better and more competitive performance regarding boundary recall, segmentation error, and achievable segmentation accuracy. It is much faster than other methods because it does not use an iterative optimization process. It performs spatial and color quantization in advance to represent pixels and superpixels. Unlike iterative approaches, it aggregates pixels into spatially and visually consistent superpixels using maximum a posteriori (MAP) estimation at pixel-level and region-level. Motivated by these works, we propose an approach that combines the USEQ superpixel resizing framework and an encoder–decoder-based segmentation network in a unified manner.

## 3. Materials and Methods

### 3.1. Datasets and Preprocessing

**Figure 1:**
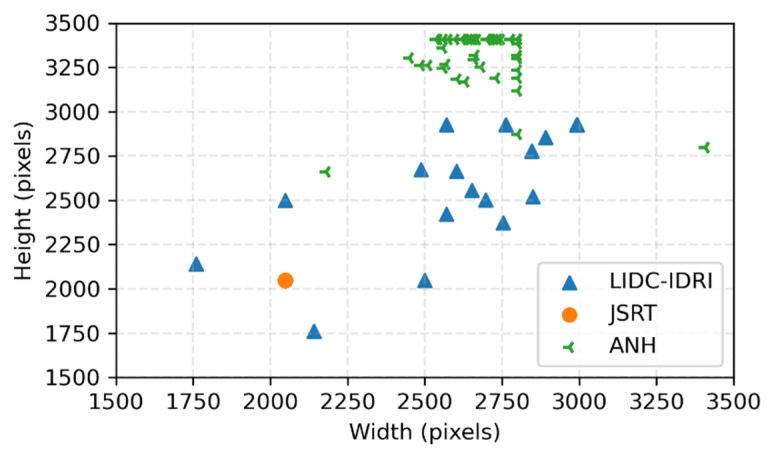
Resolution distribution of all images.

All subjects gave their informed consent for inclusion before they participated in the study. The study was conducted in accordance with the Declaration of Helsinki, and the protocol was approved by the Ethical Committee of China Medical University Hospital, Taichung, Taiwan (CMUH106-REC2-040 (FR)). The datasets used in this study include two public datasets: JSRT and Lung Image Database Consortium Image Collection (LIDC-IDRI) [37], and a non-public An Nan Hospital (ANH) dataset collected from An Nan Hospital. The images used in these three datasets are 247, 33, and 58, respectively. JSRT images have a fixed resolution of 2048 × 2048. However, the resolutions of LIDC-IDRI and ANH images are varied. The average resolutions of LIDC-IDRI and ANH are 2700 × 2640 and 2705 × 3307, respectively. The resolution distribution of all images is shown in Figure 1.

**Figure 2:**
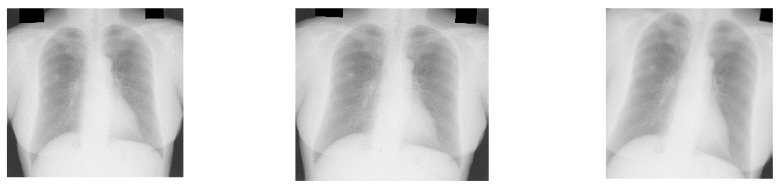
Augmented images from the JSRT dataset with different rotation degrees.

**Figure 3:**
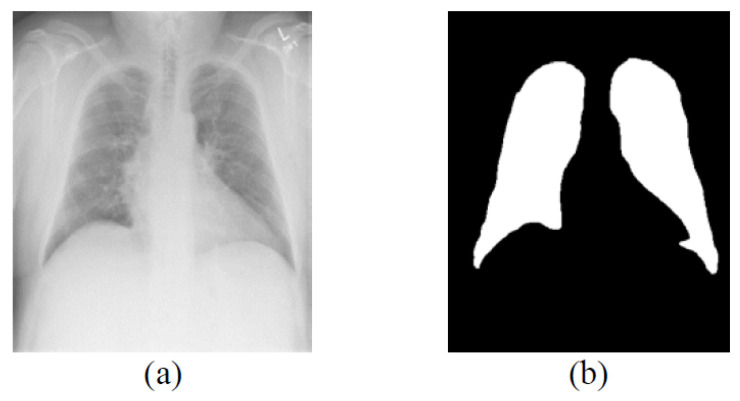
Ground truth example of the LIDC-IDRI dataset. (**a**) Original image. (**b**) Ground truth image.

The original image pixels are stored in 12-bit with 4096 graylevels. The file format for the LIDC-IDRI and ANH datasets is Digital Imaging and Communications in Medicine (DICOM), while headers are not used in the JSRT dataset. Therefore, the original images are mapped to 8-bit and stored in PNG format at their actual sizes. In most cases, deep learning algorithms perform better when trained on more data. Therefore, this work augments images by generating randomly rotated images with a maximum rotation of ± 10 degrees for each original image. Examples of augmented images from the JSRT dataset are shown in Figure 2. The work also creates manual reference segmentations drawn by medical experts for each image. The segmentation masks are labeled with values of 0 and 1, corresponding to the background and lung fields. A ground truth example of the LIDC-IDRI dataset is shown in Figure 3.

### 3.2. Overview of the Lung Field Segmentation

**Figure 4:**
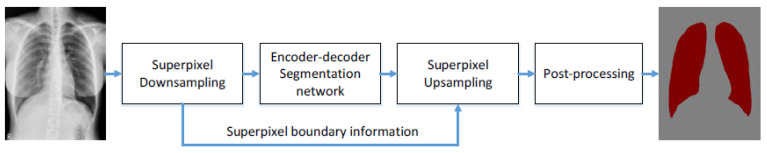
Overview of the proposed lung field segmentation method.

The proposed lung field segmentation method combines an encoder–decoder segmentation network and the USEQ superpixel resizing framework [38] to obtain high-quality segmentation results. The architecture of the method is shown in Figure 4. First, the input image is downsampled using the downsampling interpolation function to find the low-resolution image to reduce the computation time in the subsequent segmentation network. Next, the downsampled low-resolution image is processed through the encoder–decoder segmentation network to segment lung fields. Then, the proposed upsampling interpolation function upsamples the segmentation results based on the superpixel boundary information obtained from the downsampling process. The stored superpixel boundary information is used to recover the high-resolution segmentation results. Finally, post-processing is applied to correct the segmentation results.

### 3.3. USEQ Superpixel Extraction

USEQ algorithm consists of four computationally efficient steps of generating superpixels. First, it employs spatial quantization to generate the initial superpixels based on pixel locations. Second, the color space of each pixel is also quantized to obtain the dominant color within each initial superpixel. In spatial quantification, the USEQ algorithm calculates the initial width and height of a superpixel, and then defines the spatial relationship between pixels and superpixels. The initial width and height of each superpixel are computed as follows:

(1) $$w=\frac{W}{\sqrt{\delta }}$$

(2) $$h=\frac{H}{\sqrt{\delta }}$$

where *W* and *H* represent image width and height, respectively. The target number of superpixels is denoted by *δ*. Pixels belonging to superpixel $sp_{i}$ are defined as follows:

(3) $$sp_{i}=\left\{p_{k}|\left|\left|p_{k}-sp_{i}\right|\right|<\left|\left|p_{k}-sp_{j}\right|\right|\forall \;j\neq i\right\}$$

were $p_{k}$ is the position of the *k*-th pixel in an image. The spatial neighbor relationship $e\left(sp_{i},sp_{j}\right)$ between superpixels $sp_{i}$ and $sp_{j}$ is defined as follows:

(4) $$e\left(sp_{i},sp_{j}\right)=\{\begin{array}{c} 1\;\;\;\;\;\;\;sp_{i\;}\mathrm{and}\;sp_{j}\;\mathrm{are}\;\mathrm{neighbor}\;\mathrm{grid}\;\;\\ 0\;\;\;\;\;\;\;\;\;\;\;\;\;\;\mathrm{otherwise}\;\;\;\;\;\;\;\;\;\;\;\;\;\;\;\;\;\;\;\;\;\;\;\;\;\;\;\;\;\;\; \end{array}$$

These enabled the algorithm to build a spatial neighbor relationship between superpixels.

**Figure 5:**
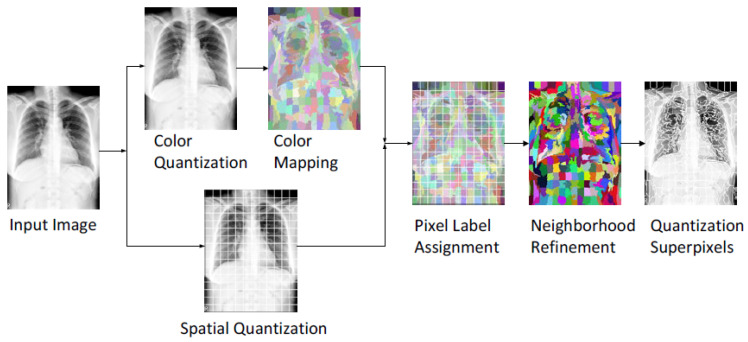
Flowchart of USEQ superpixel extraction method.

**Figure 6:**
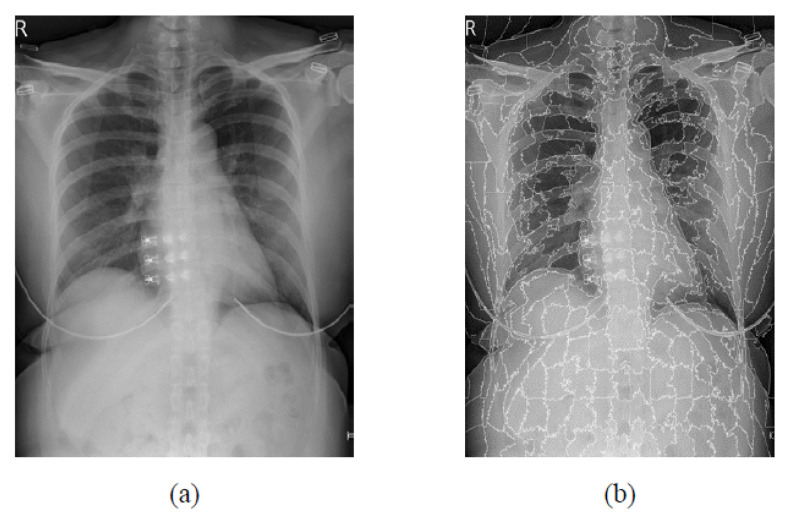
USEQ superpixel extraction result. (**a**) Input image. (**b**) Superpixel results.

Third, after the spatial and color quantifications, a non-iterative maximum a posteriori (MAP) pixel label assignment uses both spatial and color quantization results to reassign labels of pixels for better boundary adherence of objects. Finally, a MAP estimation-based neighborhood refinement is used to merge small adjacent superpixels with visual similarity to obtain superpixels with more regular and compact shapes. Figure 5 shows the flowchart of the USEQ superpixel extraction method. An example of the USEQ result is shown in Figure 6.

### 3.4. USEQ Superpixel Resizing Framework

The superpixel resizing framework is mainly composed of the downsampling interpolation function $F^{D}\left(\cdot \right)$ and the upsampling interpolation function $F^{U}\left(\cdot \right)$. Let ***I***, be the input image, and $I^{D}$ and $I^{U}$ be the downsampled and upsampled images of ***I***. The image matrix ***I*** is composed of a homogeneous matrix ***H*** of homogeneous regions and a boundary matrix ***B*** of the boundaries of objects as follows:

***I*** = ***H*** + ***B***. (5)

Here, the USEQ superpixel extraction separates the image matrix ***I*** to the homogeneous matrix ***H*** and the boundary matrix ***B***. To obtain $I^{D}$, a downsampling interpolation function $F^{D}\left(\cdot \right)$ is applied to ***I*** as follows:

(6) $$I^{D}=F^{D}(I)$$

To recover the high-resolution image $I^{U}$, the upsampling interpolation function $F^{U}\left(\cdot \right)$ is applied to $I^{D}$, and $I^{U}$ is represented as follows:

(7) $$I^{U}=F^{U}\left(I^{D}\right)$$

To obtain high-quality upsampled results which are similar to original images, the distance function $D\left(I^{U},\;I\right)$ between $I^{U}$ and ***I*** should be minimized as follows:

(8) $$D\left(I^{U},\;I\right)=min\|I^{U}-I{\|}^{2}$$

Substitute Equations (5)–(7) to Equation (8), $D\left(I^{U},\;I\right)$ is then derived as follows:

(9) $$D\left(I^{U},\;I\right)=min\|F^{U}\left(F^{D}\left(H+B\right)\right)-{\left(H+B)\right\|}^{2}.$$

For a superpixel $sp_{i}$, we classify the pixels in $sp_{i}$ into homogeneous and boundary pixels, respectively. The boundary set $sp_{i}^{B}$ of pixels in $sp_{i}$ are the pixels that spatially connected to the pixels in the neighbor superpixel $sp_{j}$, where *i* ≠ *j*, as follows:

(10) $$sp_{i}^{B}=\{p_{k}|p_{k}\in sp_{i}\;\mathrm{and}\;d(p_{k},p_{l})=1\}$$

where $d(p_{k},p_{l})=\{\;||p_{k}-p_{l}||\;|\;p_{k}\in sp_{i},p_{l}\in sp_{j},i\neq j\}$ and $p_{k}={\left[x_{k},y_{k}\right]}^{T}$ is the 2D image position of the *k*-th pixel $p_{k}$ in ***I***. The homogeneous set $sp_{i}^{H}$ is then defined as pixels are in $sp_{i}$ but are not in the boundary set $sp_{i}^{B}$ as:

(11) $$sp_{i}^{H}=sp_{i}-sp_{i}^{B}$$

With $sp_{i}^{H}$ and $sp_{i}^{B}$, $H_{M}\left(p_{k},sp_{i}\right)$ is then defined as follows:

(12) $$H_{M}(p_{k},sp_{i})=\{\begin{array}{cc} I(p_{k}), & p_{k}\in sp_{i}^{H}\\ 0, & \mathrm{otherwise} \end{array}$$

which represents the pixels in the homogeneous regions in $sp_{i}$. Similarly, $B_{M}\left(p_{k},sp_{i}\right)$ is defined as follows:

(13) $$B_{M}(p_{k},sp_{i})=\{\begin{array}{cc} I(p_{k}), & p_{k}\in sp_{i}^{B}\\ 0, & \mathrm{otherwise} \end{array}$$

which represents the pixels in the boundaries in $sp_{i}$.

Because the number of superpixels equals to the number of pixels of the downsampled image $I^{D}$, the color value $I\left(p_{i}^{D}\right)$ of the pixel $p_{i}^{D}$ of $I^{D}$ is computed from the corresponding superpixel $sp_{i}$. Therefore, downsampling interpolation function $F^{D}$(·) is designed to map the colors of pixels of $sp_{i}$ to $p_{i}^{D}$ as follows:

(14) $$F^{D}(H_{M}(p_{k},sp_{i}))=\frac{\sum_{p_{k}\in sp_{i}}^{}\left\{H_{M}(p_{k},sp_{i})|p_{k}\in sp_{i}^{H}\right\}}{\sum_{p_{k}\in sp_{i}}^{}\left\{1|p_{k}\in sp_{i}^{H}\right\}}.$$

Using Equation (14), the color value $I\left(p_{i}^{D}\right)$ of the pixel $p_{i}^{D}$ is then obtained as follows:

(15) $$I\left(p_{i}^{D}\right)=F^{D}\left(H_{M}\left(p_{k},sp_{i}\right)\right)$$

Because $sp_{i}^{H}$ contains homogeneous pixels of $sp_{i}$, the obtained $I\left(p_{i}^{D}\right)$ is also visually similar to the colors of the pixels of $sp_{i}$.

The boundary between $sp_{i}$ and $sp_{j}$ retains between $p_{i}^{D}$ and $p_{j}^{D}$ of the downsampled image, which means that the downsampling interpolation function $F^{D}$(·) can effectively preserve boundaries of objects during downsampling. In this way, we can obtain a high-quality, low-resolution image containing clear boundaries of segmented objects to avoid degradation of segmentation results. Here, $sp_{i}^{B}$ of each superpixel is reserved for boundary information and used to recover the high-resolution segmentation results during the upsampling.

To obtain the high-resolution segmentation results, the upsampling interpolation function $F^{U}\left(\cdot \right)$ is designed based on $sp_{i}^{B}$, which preserves the boundary information for superpixels in an image. Because boundary matrix ***B*** stores the boundary information during downsampling, it complements the missing boundary information for image upsampling. Thus, $F^{U}\left(\cdot \right)$ is designed to map the colors of pixels $p_{i}^{D}$ of $I^{D}$ to pixels in $I^{U}$ as follows:

(16) $$F^{U}(I(p_{i}^{D}),p_{k})=\{\begin{array}{cc} I(p_{i}^{D}), & p_{k}\in sp_{i}^{H}\\ B_{M}(p_{k},sp_{i}), & p_{k}\in sp_{i}^{B}\\ 0, & I(p_{i}^{D})\in \mathrm{background} \end{array}$$

where $I\left(p_{k}^{U}\right)=F^{U}\left(I\left(p_{i}^{D}\right),p_{k}\right)$ is the color of $p_{k}$ which belongs to the superpixel $sp_{i}$ of the image. In Equation (16), pixels of the same superpixels of the upsampled image will have consistent colors. Moreover, the colors of pixels between boundaries will differ based on the superpixel information. Thus, the upsampled image can maintain the original boundaries of segmented objects. In addition, the time complexity in the image upsampling step is very low, because only pixel value assignment is performed based on the superpixels.

### 3.5. Encoder–Decoder Segmentation Networks

**Figure 7:**
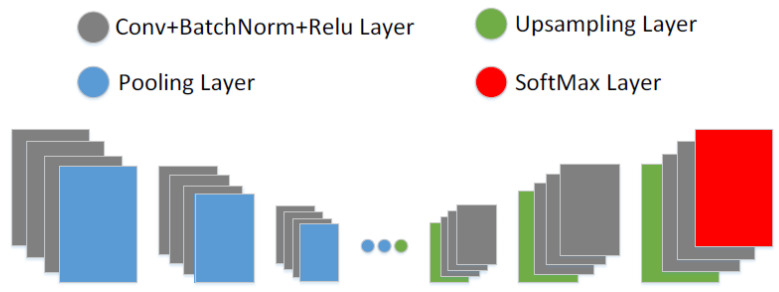
The architecture of the encoder–decoder segmentation network.

The encoder–decoder segmentation network consists of five encoder layers with corresponding decoder layers. The network architecture is shown in Figure 7. Each convolutional layer is followed by batch normalization and ReLU (rectified-linear units) nonlinearity. These are followed by max pooling with a 2 × 2 window size without overlapping and a stride length of two. The resulting feature map from max-pooling is subsampled by a factor of two which enables it to achieve translation invariance for robust classification. Although max-pooling and subsampling achieve translation invariance, they cause a loss of resolution in the feature maps. In this work, we use SegNet [24] to address this problem by storing the location of the maximum feature value in each pooling window and passing it to the decoder.

The decoder network uses the stored pooling indices to upsample the encoder′s input feature map(s). Each convolutional layer in the decoder is preceded by upsampling with the mapped pooling indices from the encoder and succeeded by batch normalization and ReLU nonlinearity. The feature map from the final decoder layer is fed to a softmax classifier for pixel-wise classification. The output of the softmax classifier is the probability of the *N*-channel image, where *N* is the number of classes. In this study, there are classes, background, and lung fields.

### 3.6. Post-Processing

The softmax classifier at the end of the decoder network classifies each pixel on the image as the background and lung fields. The purpose of this study is to segment the lungs. Because the lung field is full of air, the pixel intensity in the lung fields is very low. Therefore, the dark areas are classified as the lung fields, and the white areas on the image are classified as the background. However, due to anatomy, dark spots outside the lungs were also found in the body cavity, such as the stomach. These dark spots are also classified as lung fields. On the other hand, due to certain diseases, some white spots in the lung field are also classified as the background.

**Figure 8:**
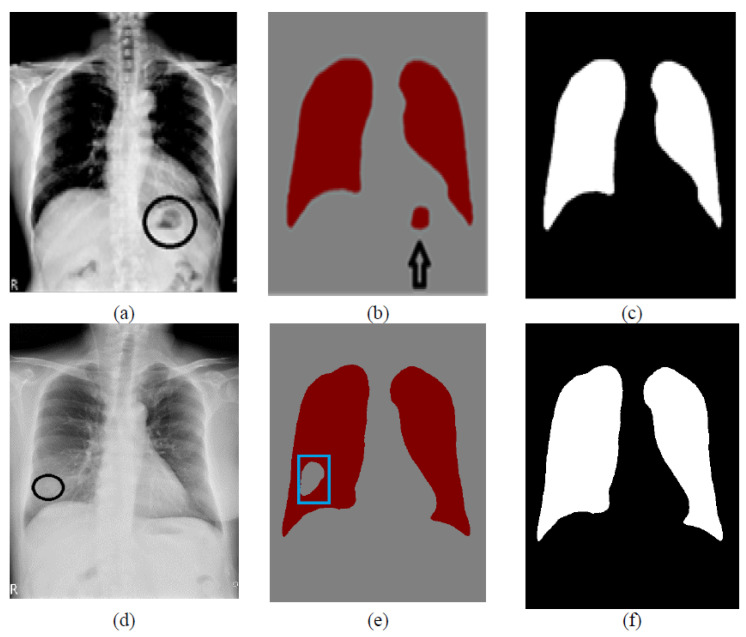
Examples of post-processing: (**a**,**d**) are the original images; (**b**) is the case of a dark spot outside the lungs which is classified as the lung; (**e**) is the case of a white spot in the lung field, which is classified as the background; (**c**,**f**) are the post-processed results.

For the above reasons, post-processing is used to correct the segmentation results. Only the two largest segmented regions are considered lung fields, the left and right lungs. Other small-segmented areas will be discarded. Similarly, the hole in the lungs will be filled. Two examples are shown in Figure 8. The entire lung filed segmentation algorithm is summarized in Algorithm 1.
**Algorithm 1**. Lung Field Segmentation**Input**: Given a set of CXR images ***X*** and a set of ground truth masks ***Y***. I∈X and M∈Y.**Output**: ***O***, the segmentation results. 1  Decompose ***I*** into homogeneous matrix ***H*** of homogeneous regions and a boundary matrix ***B*** of the boundaries of superpixels using superpixel extraction. 2  Downsample ***I*** to obtain the downsampled image $I^{D}$ using Equation (14). 3  Downsample ***M*** to obtain the downsampled image $M^{D}$.4  Store the superpixel label information for each pixel of ***I***.5  In training phase:    5.1   Input a set of $I^{D}$ and a set of $M^{D}$ to the encoder–decoder segmentation network to train the model.6  In prediction phase:    6.1   Input $I^{D}$ to the encoder–decoder segmentation network to predict the low-resolution segmentation results $O^{D}.$    6.2   Upsample $O^{D}$ to obtain the high-resolution segmentation results ***O*** using Equation (16).    6.3   Run the post-processing procedure on ***O*** to correct the segmentation results.      6.3.1    Keep the two largest regions and discard other small regions.      6.3.2    Fill all the holes in the two largest regions.7  Output the final result ***O***.

## 4. Experimental Results

### 4.1. Datasets and Model Training

The datasets used in this experiment include JSRT, LIDC-IDRI, and ANH, which consist of 247, 33, and 58, respectively. JSRT and LIDC-IDRI are public datasets, while ANH is a non-public dataset. In the experiments, we used these three datasets to train and build five segmentation network models and named each model according to the dataset. Eighty percent of each dataset is used for training and the remaining twenty percent is used for testing.

After the data argumentation process, the JSRT model is trained on 2370 JSRT images, the LIDC model is trained on 1870 LIDC-IDRI images, and the ANH model is trained on 836 ANH images. We also combine training data from the LIDC-IDRI and JSRT datasets to train on the LIDC_JSRT hybrid model. In the same way, all datasets are combined and trained on the LIDC_JSRT_ANH hybrid model. We conducted all experiments on a computer with Intel(R) Xeon(R) CPU E5-2630 v3 @ 2.40 GHz CPU and GeForce GTX TitanX GPU with 12 GB of memory. The batch size is set to 4 and the maximum number of iterations is 40,000. The Adam optimizer with learning rate of 0.0005 is used to train the network parameters. Table 1 shows the number of images used for the training and testing of each model.

### 4.2. Performance Comparison of Superpixel and Bicubic Interpolations

Since the goal of the experiments is not only to segment the lung, we also demonstrate the performance of the USEQ superpixel resizing framework. As mentioned before, the image boundaries become blurred after the downsampling and upsampling steps. Here, the peak signal-to-noise ratio (PSNR) [39] is used to evaluate the USEQ superpixel interpolation with other interpolation algorithms used in the downsampling and upsampling steps.

Given an image ***I*** with size *W* × *H* and *C* channels, image ***I*** is downsampled to image $I^{D}$ with a specified size, and then upsampled to the original resolution, which is called image $I^{U}$. Channels *C* can be ignored here because CXR images are single channel. The PSNR is defined by the mean squared error (MSE) for a single channel, the MSE is defined as follows:

(17) $$\mathrm{MSE}=\frac{1}{W\times H}\overset{W-1}{\underset{i=0}{\sum }}\overset{H-1}{\underset{j=0}{\sum }}{\left[I\left(i,j\right)-I^{U}\left(i,j\right)\right]}^{2}$$

The PSNR is defined as:

(18) $$\mathrm{PSNR}=20\cdot log_{10}\left(MAX_{I}\right)-10\cdot log_{10}\left(\mathrm{MSE}\right)$$

where the $MAX_{I}$ is the maximum value of an image. Higher PSNR means better visual quality and less information loss.

**Figure 9:**
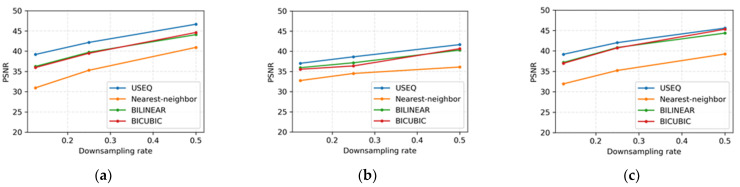
Average PSNR for different interpolation methods under different downsampling rates. (**a**) PSNR of JSRT dataset. (**b**) PSNR of LIDC-IDRI dataset. (**c**) PSNR of ANH dataset.

We compare USEQ superpixel interpolation with nearest-neighbor interpolation [40], bilinear interpolation [41], and bicubic interpolation [42] at different downsampling rates (i.e., 0.125, 0.25, and 0.5). Figure 9 shows the average PSNR evaluation results for each dataset. As shown in the figures, nearest-neighbor interpolation is the worst, bilinear and bicubic interpolations are moderate, but bicubic is slightly better. The proposed USEQ superpixel interpolation outperforms other interpolations because it considers boundary information during downsampling and upsampling.

**Figure 10:**
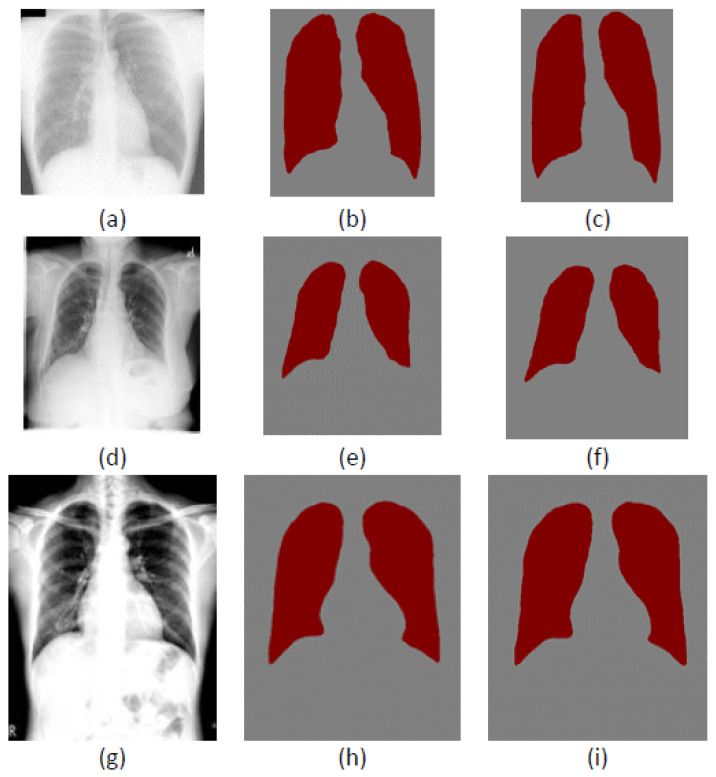
Segmentation results for two resizing algorithms: (**a**,**d**,**g**) are images from JSRT, LIDC-IDRI and ANH datasets; (**b**,**e**,**h**) are the segmentation result of the JSRT, LIDC, and ANH models using USEQ superpixel interpolation; (**c**,**f**,**i**) are the segmentation results of the JSRT, LIDC, and ANH models using bicubic interpolation.

We combine the segmentation network with different interpolation algorithms, USEQ superpixel interpolation and bicubic interpolation to evaluate the segmentation results. The segmentation network outputs are upsampled to their original space using the same interpolation algorithm used in the downsampling. Figure 10 shows segmentation results for JSRT, LIDC, and ANH models using USEQ superpixel interpolation and bicubic interpolation. Although the results are broadly similar, there are some differences in the details.

**Figure 11:**
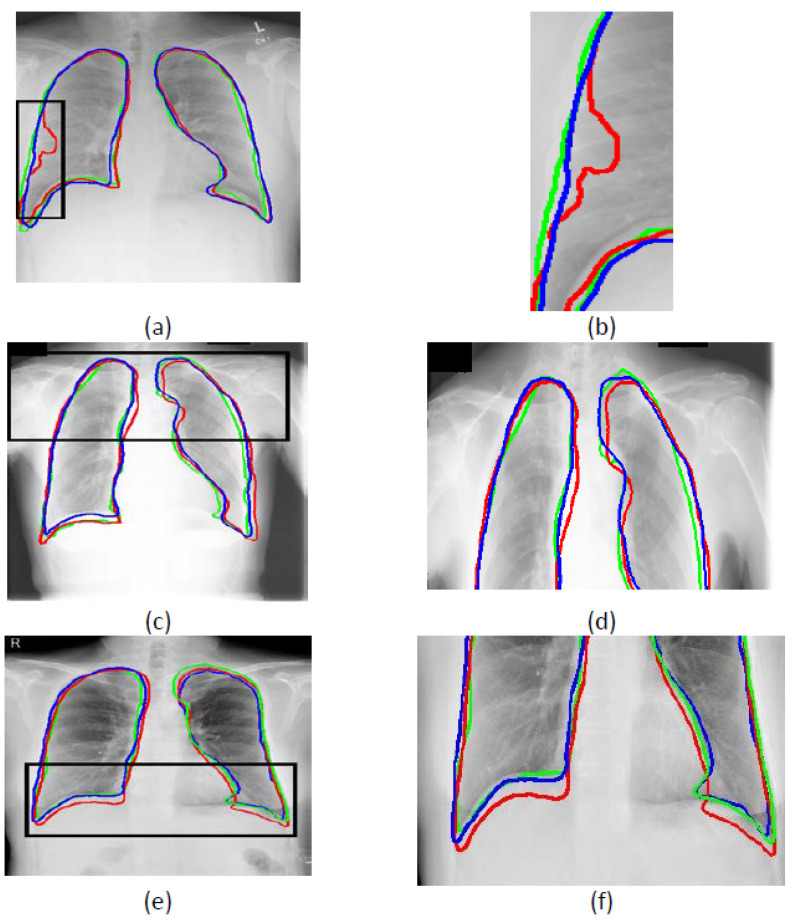
Examples of the boundary adherence between USEQ superpixel interpolation and bicubic interpolation. The blue contours are the results of USEQ, the red contours are the results of bicubic, and the green contours are the ground truth; (**a**,**c**,**e**) are the results of LIDC, JSRT, and ANH; (**b**,**d**,**f**) are the zoomed-in parts of the black rectangle in (**a**,**c**,**e**).

The contours of the segmentation results are drawn on the original image to show the adherence to the lung field boundaries. Figure 11 shows the boundary adherence between USEQ superpixel interpolation and bicubic interpolation. Figure 11a,c,e are the results of LIDC, JSRT, and ANH. Figure 11b,d,f are the zoom-in versions of it. The blue contours are the results of USEQ, the red contours are bicubic, and the green contours are the ground truth. From the zoom-in parts in Figure 11b,d,f, these figures clearly show that the contour of using the USEQ superpixel interpolation is better than that of using the bicubic interpolation.

Four metrics are also employed to measure the quantitative impact of segmentation results between USEQ superpixel interpolation and bicubic interpolation. These metrics include dice similarity coefficient (DSC), sensitivity, specificity, and Modified Hausdorff distance (MHD) as follows [10]:

(19) $$\mathrm{DSC}=\frac{2\times TP}{2\times TP+FP+FN}$$

(20) $$Setsitivity=\frac{TP}{TP+FN}$$

(21) $$Specificity=\frac{TN}{TN+FP}$$

where *TP* (true positives) represents correctly classified lung pixels, *FP* (false positives) represents pixels classified as lung but are background, *FN* (false negatives) represents pixels classified as background but are part of the lung, and *TN* (true negative) represents correctly classified background pixels. The MHD calculates the average distance between the segmentation result and the ground truth, defined as follows:

(22) $$h\left(S_{seg},S_{gold}\right)=\frac{1}{\left|S_{gold}\right|}\underset{q\in S_{gold}}{\sum }min\left\{d\left(p,q\right)|p\in S_{gold}\right\}$$

where $S_{seg}\;\mathrm{and}\;S_{gold}$ represent segmentation results and the ground truth, respectively. $\left|S_{gold}\right|$ is the total number of pixels in the ground truth. *p* and *q* are points on the boundaries of $S_{seg}$ and $S_{gold}$, and *d*(*p*,*q*) is the minimum distance of a point *p* on the boundary $S_{seg}$ to the point *q* on the boundary $S_{gold}$.

The average metric score results for each dataset are shown in Table 2. According to the metric results, USEQ superpixel interpolation has been shown to outperform bicubic interpolation. In USEQ superpixel interpolation, the average DSC, sensitivity, and specificity score is greater than 97%. The average MHD of USEQ is less than 2, while the average MHD of bicubic interpolation is about 4. Therefore, USEQ superpixel interpolation has a better boundary adherence than bicubic interpolation.

### 4.3. Cross-Dataset Generalization

**Figure 12:**
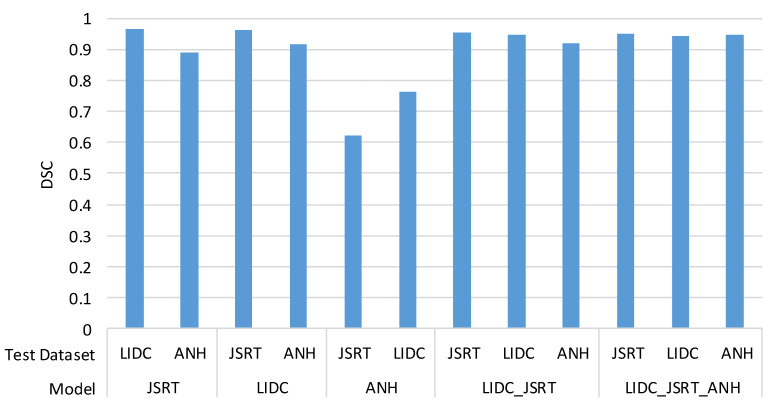
Cross-dataset test results of the DSC metric.

To test the generalization of the segmentation models, the five trained models were tested on different datasets that did not appear during their training. The cross-dataset test results of the DSC metric are shown in Figure 12. Compared with their datasets, the segmentation performance on different datasets has decreased slightly. This is because the three datasets differ in the image size and gray-level range, especially the aspect ratio (width/height). The aspect ratio of JSRT is 1, the ratio of LIDC-IDRI is about 1.02, but the ratio of ANH is 0.82. In Figure 12, except for the ANH model, the DSC scores of the other four models are approximately or higher than 90%. One possibility that may cause this situation is the difference in X-ray imaging machines.

Nevertheless, the models LIDC_JSRT and LIDC_JSRT_ANH trained from the combination of datasets have achieved excellent results. The results show that increasing data diversity can enhance the model generalization and improve performance.

### 4.4. Comparison with other Lung Segmentation Methods

Jaccard index (Ω) [3,4,11,12,13,43,44] and the mean boundary distance (MBD) are also calculated as additional metrics to compare the proposed method with other lung segmentation methods. Jaccard index is computed as:

(23) $$Ω=\frac{TP}{TP+FP+FN}$$

MBD measures the average distance between the boundary *S* of the segmentation result and the boundary *T* of the ground truth, defined as follows [4]:

(24) $$\mathrm{MBD}=\frac{1}{2}\left(\frac{\sum_{i}d\left(s_{i},T\right)}{\left|\left\{s_{i}\right\}\right|}+\frac{\sum_{i}d\left(t_{j},S\right)}{\left|\left\{t_{j}\right\}\right|}\right)$$

where $s_{i}$ and $t_{j}$ are the points on boundaries *S* and *T*, respectively. $d\left(s_{i},T\right)$ is the minimum distance of point $s_{i}$ on boundary S to boundary T, defined as follows:

(25) $$d\left(s_{i},T\right)={min}_{j}\|s_{i}-t_{j}\|$$

Comparisons are made only on the JSRT dataset, as most studies use this dataset, as listed in Table 3. The proposed method outperforms other methods, such as SEDUCM [4], SIFT-Flow [43], and MISCP [44] in the Jaccard index, DSC, and MDB metrics. It also excels in the variance of the Jaccard index and DSC metrics. Variance is understood in machine learning as how much the prediction for a given point varies between model implementations. Bias measures the overall gap between the model′s predictions and the ground truth. In some cases, low variance does not guarantee a model with low bias. However, the Jaccard index measures the overlap between model predictions and ground truth in semantic segmentation. Therefore, the higher the value of Ω, the less biased the model predictions are. The bias-variance trade-off in our method is minimized compared to other methods. That is, our method has the most consistent prediction results.

Although some methods in the literature slightly outperform the proposed method in terms of computational time, our method is still comparable due to the different computing power of machines. The computational time of the methods was computed on images of size 256 × 256. The proposed method achieves an average time score of 4.6 s on CPU and 0.02 seconds on GPU. According to the results described in Table 3, our method outperforms other methods on Ω, DSC, and MBD. Considering the difference in the computing power of machines, the speed of this method is not bad compared to other methods.

## 5. Conclusions

We propose lung field segmentation in this study using the USEQ superpixel resizing framework and an encoder–decoder segmentation network. The superpixel resizing framework stores the superpixel boundary information in the downsampling step and reloads the boundary information in the upsampling step. In this way, the framework can reduce information loss during downsampling and reconstruct the boundaries of segmentation results during upsampling. Using the superpixel resizing framework, the computation time of the segmentation network can also be reduced while preserving the quality of the segmentation.

This study uses the ability of superpixels to adhere to object boundaries. USEQ generates superpixels based on spatial and color quantization results to reveal the boundaries of objects in an image perceptually. It uses this information during image resizing to maintain the resolution and the correct localization of objects. This property enables the proposed method to delineate lung fields in CXR images accurately. To evaluate the impact of segmentation results between USEQ superpixel interpolation and bicubic interpolation, four metrics, DSC, sensitivity, specificity, and MHD, are used. The experimental results show that the USEQ superpixel interpolation has better results on all metrics in the three datasets. The proposed method is also compared with existing methods on the JSRT dataset. Our method not only outperforms other methods in Jaccard index, DSC, and MDB metrics, but also performs better on the bias-variance trade-off. That is, our method has the most consistent prediction results. Cross-dataset evaluations are also performed. The results show that increasing data diversity can enhance the model generalization and improve performance.

To conclude, the proposed method is the first to provide a superpixel resizing framework for lung field segmentation. Our approach can be used for the analysis of CXR lung fields. The technique can potentially be extended to other medical image segmentation problems to reduce computation time and preserve segmentation quality.

## Figures and Tables

**Table 1:** The number of images used for training and testing of each model.

Models	Training Data	Testing Data	Total
JSRT	2370	594	2964
LIDC	1870	462	2332
ANH	836	208	1044
LIDC_JSRT	4240	1054	5294
LIDC_JSRT_ANH	5076	1262	6338

**Table 2:** Quantitative performance of the segmentation results between USEQ superpixel interpolation and bicubic interpolation.

Models	Lungs	USEQ Superpixel Interpolation				Bicubic Interpolation			
		DSC	Sensitivity	Specificity	MHD	DSC	Sensitivity	Specificity	MHD
JSRT	Left	0.977	0.973	0.996	1.107	0.953	0.949	0.992	2.779
	Right	0.978	0.975	0.999	1.002	0.96	0.958	0.992	4.201
LIDC	Left	0.972	0.971	0.995	0.888	0.926	0.93	0.989	4.645
	Right	0.972	0.97	0.994	1.718	0.938	0.934	0.989	6.72
ANH	Left	0.97	0.979	0.999	1.942	0.953	0.936	0.994	4.428
	Right	0.982	0.978	0.994	1.24	0.948	0.949	0.992	3.783
LIDC_JSRT	Left	0.973	0.966	0.996	0.815	0.95	0.941	0.994	3.284
	Right	0.979	0.978	0.995	1.448	0.948	0.941	0.991	3.755
LIDC_JSRT_ANH	Left	0.964	0.967	0.994	1.736	0.947	0.962	0.991	2.962
	Right	0.968	0.975	0.994	2.094	0.952	0.953	0.992	3.81
Average		0.9735	0.9732	0.9956	1.399	0.9475	0.9453	0.9916	4.0367

**Table 3:** Comparison of lung field segmentation methods on JSRT dataset.

Method	Ω (%)	DSC (%)	MBD (mm)	Time (s)
Proposed method	95.5 ± 0.02	97.7 ± 0.01	0.542 ± 0.79	CPU: 4.6 GPU:0.02
SEDUCM [4]	95.2 ± 1.8	97.5 ± 1.0	1.37 ± 0.67	<0.1
SIFT-Flow [43]	95.4 ± 1.5	96.7 ± 0.8	1.32 ± 0.32	20∼25
MISCP [44]	95.1 ± 1.8	/	1.49 ± 0.66	13∼28
Hybrid voting [12]	94.9 ± 2.0	/	1.62 ± 0.66	>34
Local SSC [13]	94.6 ± 1.9	97.2 ± 1.0	1.67 ± 0.76	35.2
Human observer [12]	94.6 ± 1.8	/	1.64 ± 0.69	/
GTF [11]	94.6 ± 2.2	/	1.59 ± 0.68	38
InvertedNet [3]	94.6	97.2	0.73	7.1
PC post-processed [12]	94.5 ± 2.2	/	1.61 ± 0.80	30
ASM tuned [12]	92.7 ± 3.2	/	2.30 ± 1.03	1
ASM_SIFT [12]	92.0 ± 3.1	/	2.49 ± 1.09	75
AAM whiskers [12]	91.3 ± 3.2	/	2.70 ± 1.10	3

The values on the table are recorded as mean ± standard deviation except for the time column.

## Data Availability

For detailed information of image data availability for research purposes, please contact the corresponding authors.
